# Risk of osteoporotic fracture after steroid injections in Medicare patients

**DOI:** 10.1186/1748-7161-9-S1-O46

**Published:** 2014-12-04

**Authors:** Leah Carreon, Kevin Ong, Edmund Lau, Steven Kurtz, Steven Glassman

**Affiliations:** 1 Norton Leatherman Spine Center, Louisville, Kentucky, USA; 2 Exponent, Philadelphia, Pennsylvania, USA; 3 Exponent, Menlo Park, California, USA

## Background

A recent study showed that lumbar epidural steroid injections increased osteoporotic spine fracture risk.

## Aim

We further evaluated associations between steroid injections and osteoporotic fracture risk by analyzing the Medicare database and including large joint and transforaminal steroid injections; as well as osteoporotic hip and wrist fractures. A systemic effect would increase risk in all fracture locations regardless of injection site. A local effect would result in a disproportionate increased risk of spine fractures when steroids were injected into the spine.

## Design

Epidemiological study using national administrative data.

## Methods

Patients who had a steroid injection into the epidural space (ESI), transforaminal space (TSI) or large joint (LJSI) were identified. Patients younger than 65 and those diagnosed with a prior osteoporotic fracture were excluded. Patients were followed continuously until fracture, withdrawal from Medicare or death. Kaplan-Meier survival and Cox regression analysis were performed to determine adjusted fracture risk (Adjusted Hazard Ratio (HR)) for each type of injection, accounting for patient characteristics such as age, sex, Charlson Comorbidity Index (CCMI), Cushing’s syndrome or long term steroid use.

## Results

Osteoporotic spine fracture risk after ESI, TSI or LJSI was influenced by age, race, sex and CCMI. The risk decreases with each additional ESI (HR=0.98), TSI (HR=0.99) or LSJI (HR=0.96). Long term steroid use increased spine fracture risk in ESI (HR=1.86) and LJSI patients (HR=1.41). Osteoporotic hip fracture risk was influenced by age, race, sex, CCMI, Cushing’s disease, LJSI (HR=0.95) and TSI (HR=0.95), but not ESI. Osteoporotic wrist fracture risk was influenced by age, race, sex, CCMI, LJSI (HR=0.91), but not by ESI or TSI.

## Conclusions

Analysis of patients in the Medicare database showed that ESI, TSI or LJSI decreased osteoporotic spine fracture risk; but this may not be clinically relevant. Successive ESIs did not influence osteoporotic hip or wrist fracture risk, while LJSIs reduced the risk. Prolonged steroid exposure increases spine fracture risk in ESI and LJSI patients. Acute exposure to exogenous steroids via the epidural space, transforaminal space or large joints does not seem to increase the risk of an osteoporotic fracture of the spine, hip or wrist.

